# The crosstalk between glutathione metabolism and non-coding RNAs in cancer progression and treatment resistance

**DOI:** 10.1016/j.redox.2025.103689

**Published:** 2025-05-19

**Authors:** Lu Chang, Chao Qin, Jianbo Wu, Haoqin Jiang, Qianqian Xu, Jian Chen, Xiao Xu, Xinju Zhang, Ming Guan, Xuan Deng

**Affiliations:** Department of Laboratory Medicine, Huashan Hospital Fudan University, Shanghai, 200040, China

**Keywords:** Glutathione metabolism, miRNAs, lncRNAs, Cancer progression, Resistance

## Abstract

Excessive reactive oxygen species (ROS) are closely associated with the initiation and progression of cancers. As the most abundant intracellular antioxidant, glutathione (GSH) plays a critical role in regulating cellular ROS levels, modulating physiological processes, and is intricately linked to tumor progression and drug resistance. However, the underlying mechanisms remain not fully elucidated. Non-coding RNAs (ncRNAs), including long non-coding RNAs (lncRNAs) and microRNAs (miRNAs), are key regulators of GSH levels. Different ncRNAs modulate various pathways involved in GSH metabolism, and these regulatory targets have the potential to serve as therapeutic targets for enhancing cancer treatment. In this review, we summarize the functions of GSH metabolism and highlight the significance of ncRNA-mediated regulation of GSH in cancer progression, drug resistance, and clinical applications.

## 1 Introduction

Reactive oxygen species (ROS), i.e., Superoxide (O_2_•^-^), Hydrogen Peroxide (H_2_O_2_), Peroxynitrite (ONOO^−^) and Hydroxyl Radical (•OH), generally considered by-products of cellular aerobic respiration, are formed by partial reduction of molecular oxygen [1,2]. A small amount of ROS is necessary for maintaining physiological activities of normal cells [3,4]. Elevated levels of ROS, exceeding the clearance capacity of non-malignant cells, however, resulting in accumulation of oxidized DNA, proteins and lipids [1,5]. This is lethal to cells. ROS exhibits distinct and intricate effects in non-malignant and malignant cells [6,7]. Accumulation of ROS has been reported in different forms of cancer caused by genetic instability, mitochondrial dysfunction and altered metabolism pathways in cancer cells [8]. These compounds trigger activation of pro-survival pathways, loss of tumor suppressor gene functions, adaptation to hypoxia, and carcinogenic mutations, creating favorable conditions for cancer survival [9].

Glutathione (GSH), the predominant non-protein thiol found in mammalian tissues, plays a dual role in cancer biology [10]. As a crucial intracellular antioxidant, GSH controls the cellular redox balance, safeguarding cells against damage caused by ROS, lipid peroxides, nitrogen species, and foreign chemicals [11]. However, in the context of cancer, the roles of GSH extend beyond protection. It is implicated in tumor initiation, progression, and the modulation of treatment responses. Higher levels of GSH in tumor cells have been linked to enhanced tumor growth and increased resistance to chemotherapy [12,13]. This paradoxical role of GSH in both protecting healthy cells and facilitating tumor survival and drug resistance highlights the complexity of its functions in cancer biology [8]. Novel therapeutic strategies are being designed to target the GSH-based antioxidant defense in tumors, with the goal of enhancing treatment efficacy and reducing drug resistance [14]. These approaches include direct targeting of GSH, indirect interventions, and the use of GSH-based prodrugs [15].

GSH metabolism is precisely regulated by uptake of GSH precursors, GSH biosynthesis, and degradation [16]. In addition to stimulation by xenobiotics, the aberrant expression of both coding and non-coding genes during tumor progression can lead to alterations in GSH metabolic intermediates, transporters, and catalytic enzymes, ultimately resulting in the reprogramming of GSH metabolism. Non-coding RNAs (ncRNAs) are RNA transcripts with limited protein-coding potential and could be divided into short and long ones based on their lengths [17]. The short ncRNAs are these molecules less than 200 nucleotides in length, including microRNAs (miRNAs) and small nucleolar RNAs (snoRNAs), etc. [18,19]. Long ncRNAs (lncRNAs) usually refer to transcripts that are more than 200 nucleotides [20]. Circular RNAs (circRNAs) represent as another subtype of ncRNA with covalently closed loop structure, although increasing evidence showing that some circRNAs are translated under conditions that favor cap-independent translation [21]. These ncRNAs are frequently dysregulated in a variety of diseases, especially in cancer, and have emerged as crucial modulators that fine-tune GSH homeostasis through regulation of metabolic enzymes and transporters. The intricate interplay among these ncRNAs in GSH-related pathways underscores their potential as targets for innovative cancer therapies. In this review, we discuss the role of ncRNAs in aberrant GSH metabolism in cancers and anti-cancer strategies through targeting GSH-ncRNA interactions.

## 2 Regulation of GSH in tumor progression and drug resistance

### 2.1 GSH metabolism

GSH, a tripeptide, is synthesized from glycine, glutamate, and cysteine. The amino acid cysteine serves as the rate-limiting precursor for GSH synthesis and confers its antioxidant properties [22]. For most cells, cysteine is sourced extracellularly and delivered intracellularly via transporters. A particular antiporter system called Xc- (comprising the heavy chain subunit SLC3A2 and the light chain subunit SLC7A11) intakes oxidized cystine and outputs glutamate [23]. Reduced cysteine may be imported by other non-specific cation transporters such as ASCT1/2 [24]. Cells in certain tissues have the ability to convert methionine into cysteine through the transsulfuration pathway [25]. Glutamine is actively incorporated by cells via transporters such as ASCT2 and deaminated by enzymes called glutaminases (GLS) or transaminases [26]. Serine, threonine, and other amino acids can be used to produce glycine, the third component of GSH [27].

GSH biosynthesis occurs in the cytosol and involves two ATP-dependent enzymatic reactions [28]. The initial step involves the formation of the dipeptide γ-glutamylcysteine (γ-GCS), formed by the linkage of glutamate and cysteine. This rate-limiting process is catalyzed by glutamate-cysteine ligase (GCL) [29]. GCL comprises two subunits: the catalytic subunit (GCLC) and the modifier subunit (GCLM). The next step, the final synthesis of GSH, is catalyzed by glutathione synthetase (GSS).

γ-Glutamyltransferase (GGT) is a membrane-bound enzyme that starts the extracellular breakdown of GSH [30]. Dipeptidases on the cell surface further break down cystinylglycine into cysteine and glycine. This promotes GSH synthesis by offering its constituent glutamate and cysteine, a process known as the γ-glutamyl cycle [31]. The multidrug resistance protein (MRP) also transports GSH and its conjugates out of cells for elimination [32]. Intracellular GSH can be degraded by enzymes, including ChaC glutathione-specific γ-glutamylcyclotransferase 1 (ChaC1) and the defective glutathione oxidase (DUG) enzymes [33]. In contrast to the membrane attached GGT, the DUG and ChaC1 enzymes were unexpectedly located in the cytoplasm.

Under physiological conditions, reduced GSH predominates at concentrations 10-100-fold higher than its oxidized form, glutathione disulfide (GSSG) [34]. During oxidative stress, GSH is most frequently found to be elevated [35]. It serves as a critical cofactor for glutathione peroxidases (GPXs) and glutathione S-transferases (GSTs) in neutralizing ROS and other free radicals, thereby protecting cellular components from oxidative damage [36]. During this process, GPX utilizes monomeric GSH as an electron donor and reduces H_2_O_2_ to water while generating GSSG, which is subsequently recycled back to GSH by glutathione reductase (GR) and thioredoxin reductase (TrxR) in NADPH-dependent reactions [37]. This continuous interconversion constitutes the GSH/GSSG redox pair, working in concert with other redox systems (FAD/FADH2 and NAD(P)/NAD(P)H), thereby maintaining dynamic cellular redox equilibrium [22]. The GST superfamily, including cytosolic (e.g., GSTP1) and microsomal (MGSTs) isoforms, further expands GSH's protective role by catalyzing conjugation reactions with diverse endogenous and xenobiotic electrophiles [38]. Additionally, GSH participates in detoxification pathways, serving as a cofactor for the glyoxalase system to convert cytotoxic methylglyoxal (a glycolysis byproduct) to d-lactate [39] and supporting formaldehyde dehydrogenase in mitigating formaldehyde-induced DNA damage [40].

**Fig. 1:**
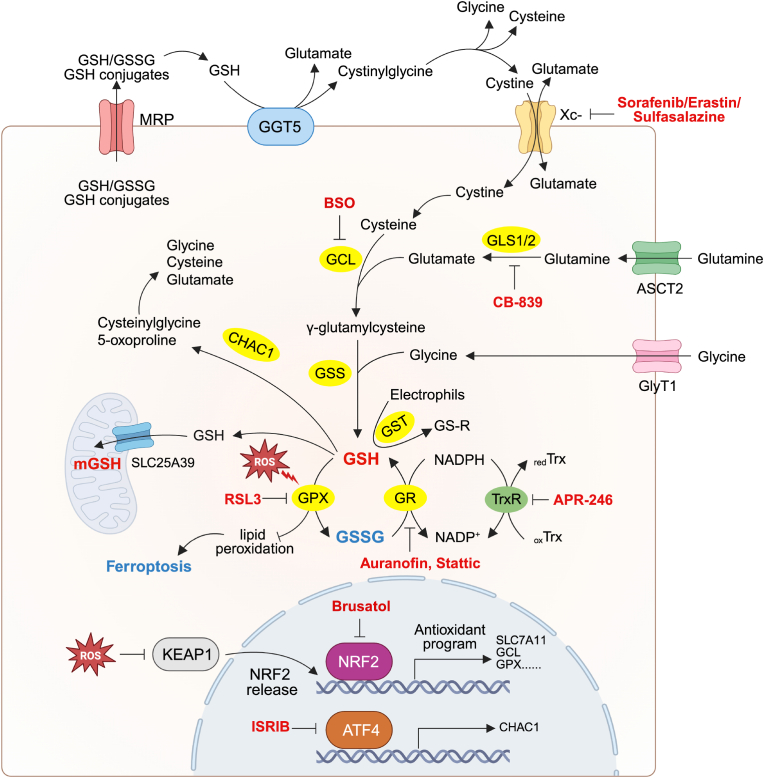
Dynamic regulation of GSH metabolism in cancer The intricate processes involved in the synthesis, uptake, and degradation of the tripeptide GSH, which is composed of cysteine, glutamate, and glycine, are illustrated. Cystine is imported into cells via the Xc-antiporter (comprising the heavy chain subunit SLC3A2 and the light chain subunit SLC7A11) in exchange for glutamate. Glutamine is actively taken up transporters ASCT1/2 and deaminated by GLS1/2 to produce glutamate. Serine, threonine, along with other amino acids, contribute to glycine production. GSH biosynthesis occurs in the cytosol, beginning with the formation of γ-glutamylcysteine from glutamate and cysteine, catalyzed by GCL. GCL consists of a catalytic subunit (GCLC) and a modifier subunit (GCLM). The second step is the synthesis of GSH from γ-glutamylcysteine and glycine, catalyzed by glutathione synthetase (GSS). Extracellular GSH is degraded by GGT, producing glutamate and cysteinylglycine, which are further broken down into cysteine and glycine. Intracellular GSH is degraded by enzymes such as ChaC1. CHAC1 is transcriptionally activated by ATF4. GSH undergoes oxidation to form GSSG (GSH disulfide) by GPX and is reduced back to GSH by GR and TrxR using NADPH, maintaining cellular redox balance. GSH also conjugates with harmful compounds by GST, facilitated by transporters like MRP, to protect against oxidative stress. Cytosolic GSH enters mitochondria via direct transporters such as SLC25A39. When under ROS stress, NRF2 protein is released from KEAP1 and translocates into nucleus, increased transcription of antioxidant genes including SLC7A11, GCL, GPX. Inhibitors targeting GSH metabolism enzymes, transporters and transcription factors are also demonstrated (Sorafenib/Sulfasalazine/Erastin for Xc-, BSO for GCL, Auranofin and Stattic for GR, RSL3 for GPX4, Brusatol for NRF2, ISRIB for ATF4, APR-246 for TrxR, CB-839 for GLS). **Abbreviation:** GSH, Glutathione; Xc-, Cystine/glutamate antiporter; SLC7A11, Solute Carrier Family 7 Member 11; SLC3A2, Solute Carrier Family 3 Member 2; ASCT2, Alanine-Serine-Cysteine Transporter 2; GlyT1, Glycine Transporter 1; GLS1/2, Glutaminase 1/2; GCL, Glutamate Cysteine Ligase; GCLC, Glutamate Cysteine Ligase Catalytic Subunit; GCLM, Glutamate Cysteine Ligase Modifier Subunit; GSS, Glutathione Synthetase; GST, glutathione S-transferase; GGT, γ-Glutamyl Transpeptidase; CHAC1, ChaC Glutathione-Specific γ-Glutamylcyclotransferase 1; ATF4, Activating Transcription Factor 4; GSSG, Glutathione Disulfide; NADPH, Nicotinamide Adenine Dinucleotide Phosphate (reduced form); TrxR, Thioredoxin Reductase; oxTrx, Oxidized Thioredoxin; redTrx, Reduced Thioredoxin; GR, Glutathione reductase; MRP, Multidrug Resistance-associated Protein; SLC25A39, Solute Carrier Family 25 Member 39; ROS, Reactive Oxygen Species; NRF2, Nuclear Factor Erythroid 2-Related Factor 2; KEAP1, Kelch-like ECH-associated Protein 1; GPX, Glutathione Peroxidase; BSO, Buthionine Sulfoximine; GR, Glutathione Reductase.

The majority (∼85 %) of cellular GSH is located in the cytoplasm, mainly in its reduced form [41]. Mitochondrial GSH (mGSH) accounts for approximately 15 % of cellular GSH but with a higher concentration due to the lower matrix volume [42]. Cytosolic GSH enters the mitochondrial inner membrane via transporters, including the 2-oxoglutarate carrier (OGC; SLC25A11) and the dicarboxylate carrier (DIC; SLC25A10) [43]. Proteomic analysis revealed two additional GSH transporter candidates, SLC25A39 and SLC25A40 [44]. Levels of both proteins are regulated by GSH availability. Silencing of SLC25A39/40 expression led to reduced levels of GSH and GSSG in mitochondria (Fig. 1).

### 2.2 GSH in tumor initiation and progression

GSH and other antioxidants can be protective and pathogenic when considering the dual roles ROS play in cancer. To be specific, GSH safeguards cells from pro-neoplastic oxidative stress [45]. On the other hand, the antioxidant defense and detoxification of carcinogens by GSH are of utmost importance for maintaining cancer cell survival. Dysregulation of GSH metabolism has been identified in most types of malignancies. This is because genes involved in GSH uptake, synthesis, regeneration, and utilization are under the transcriptional regulation of oncogenic signaling pathways. The Kelch-like ECH-associated protein 1/nuclear factor erythroid 2-related factor 2 (KEAP1/NRF2) cascade primarily drives resistance to oxidative damage [46]. It activates expression of genes including SLC7A11, GCL, GPX and engages in antioxidant responses.

Harris et al. demonstrated that GSH production, driven by GCLM, was crucial for cancer initiation [47]. The onset of PI(3)K/Akt-driven breast cancer spheroids also requires enhanced GSH biosynthesis [48]. It is reported that multiple myeloma (MM) uptakes external glycine for GSH production via channel protein SLC6A9, which benefits MM cell proliferation [49]. Inhibition of SLC7A11 resulted in cessation of GSH synthesis and targeted cell death in KRAS-mutant cancer cells, leading to the prevention of tumor development in *in vivo* models [23].

These mechanistic insights collectively underscore how cancer cells hijack antioxidant systems for survival, raising important questions about the role of dietary antioxidants in tumor biology. The relationship between dietary antioxidants and cancer progression exhibits complex context-dependent characteristics [50]. Although antioxidants (e.g., N-acetylcysteine or vitamin E) are commonly marketed as health supplements, emerging evidence suggests they may accelerate cancer dissemination by stabilizing pro-metastatic transcription factors like BACH1 [51,52]. Conversely, restriction of antioxidant precursors (e.g., cysteine, glycine) can induce cancer cell death and suppress tumor growth [53,54]. These findings suggest the need for caution when using dietary supplements containing antioxidants.

### 2.3 GSH in cancer treatment resistance

Cancer cells have developed diverse strategies to avoid death or suppress death-inducing signals to become drug tolerant. Increased concentrations of GSH could provide protection against oxidative stress induced by chemotherapy and radiotherapy, promoting resistance to these treatments. It is demonstrated by Zhang et al. that AKR1B1/STAT3 interaction causes a rise in the expression of SLC7A11. As a result, GSH de novo synthesis is boosted, and this helps in ROS removal and resistance to EGFR TKI treatment [55]. GSH metabolism dysregulation is also shown to induce resistance to neoadjuvant chemotherapy (NAC) using cisplatin-based drugs for bladder cancer [56]. These insights into GSH's multifaceted role in cancer therapy resistance not only highlight its direct interaction with therapeutic agents but also its impact on cell signaling and antioxidative defense, offering a theoretical basis for developing novel anti-cancer strategies targeting GSH metabolism.

Ferroptosis, a unique type of controlled cell death, is caused by the accumulation of iron-dependent lipid peroxides [57]. It functions as an inherent tumor suppressor and actively contributes to tumor biological processes [58]. To prevent ferroptosis and promote tumor progression, cancer cells have developed essential mechanisms to activate anti-ferroptotic systems. Glutathione peroxidase 4 (GPX4) is one of the most important defensive antioxidant systems of ferroptosis. GSH could act as a cofactor of GPX4. It facilitates GPX4 activation and reduces lipid peroxides on cell membranes and protecting cells against ferroptosis [59,60]. For instance, tumor suppressor p53 inactivation or oncogenic KRAS activation mediates the heightened activity of the SLC7A11/GSH/GPX4 pathway, driving tumor growth and enhancing treatment resistance [61,62].

A crucial function of GSH is also being recognized for its role in the tumor microenvironment. In solid tumors, cancer cells are surrounded and supported by other cells (namely fibroblasts, infiltrating immune cells and endothelial cells), extracellular vesicles, chemicals, and blood vessels, known as tumor microenvironment. Evidence shows that GSH was crucial for effector T cell response and proliferation [63]. It was demonstrated by Kurniawan et al. that GSH catalyzed by GCLC is essential for the anti-tumor role of regulatory T cells (Tregs) [64]. Ovarian cancer cell-derived plasma gelsolin, which is transported by extracellular vesicles (EVs), induces apoptosis of CD8+T cells [65]. This could in turn lead to enhanced GSH production and resistance to cisplatin in cancer cells. Cancer associated fibroblasts (CAFs) could enhance extracellular cysteine supply to facilitate GSH production of pancreatic ductal adenocarcinoma (PDAC) cells [66]. This results in resistance to ferroptosis. Nevertheless, studies elucidating GSH metabolism reprogramming in other components of the tumor microenvironment are still lacking.

### 2.4 GSH metabolism as a tumor therapeutic target

As discussed above, GSH has functions in promoting cancer initiation, progression and treatment resistance, making it a promising target for cancer treatment. Studies have been conducted to discover inhibitors of key regulators involved in GSH metabolism (Table 1).

**Table 1:** Inhibitors targeting GSH Metabolism Key Enzymes and Regulatory Genes.

Enzyme/Regulatory Gene	Function	Inhibitor(s)	Ref
GCL	Catalyzes the rate-limiting step in GSH synthesis, forming γ-glutamylcysteine	BSO: Blocks γ-glutamylcysteine synthesis	[73]
NRF2	A key transcription factor regulating antioxidant response genes, including GCL, GST, GPX, and GR	Brusatol: Inhibits NRF2 activity	[78]
GLS	Converts glutamine to glutamate	CB-839: GLS1/2 antagonist	[77]
GR	Reduces GSSG to GSH, maintaining redox balance	Auranofin	[79]
		Stattic	[80]
ATF4	Transcription factor that regulates stress response genes, including GSH synthesis enzymes	ISRIB: Inhibits ATF4	[82]
Xc-	Providing cysteine as a key precursor for GSH synthesis	Sorafenib/Sulfasalazine/Erastin: Inhibits system xc-	[84,85]
GPX	Uses GSH to reduce H_2_O_2_ and organic peroxides	(1S,3R)-RSL3 and ML162/ML210: Sec46 covalent inhibitor	[91]
TrxR	Reduces thioredoxin	APR-246: Promotes GSH efflux	[94]
Enzymes containing flavin	Maintenance of intracellular REDOX balance	DPI: Inhibits enzymes	[95]

GCL, Glutamate-cysteine ligase; GSH, Glutathione; BSO, Buthionine sulfoximine; NRF2, Nuclear factor erythroid 2-related factor 2; GCL, Glutamate-cysteine ligase; GST, Glutathione S-transferases; GPX, Glutathione peroxidase; GR, Glutathione reductase; GLS, Glutaminase; ATF4, Activating transcription factor 4; ISRIB, Integrated stress response inhibitor; Xc-, System cystine/glutamate antiporter; (1S,3R)-RSL3, (1S,3R)-2-(2-chloroacetyl)-2,3,4,9-tetrahydro-1[4(methoxycarbonyl)phenyl]-1H-pyrido [3,4-b]indole-3-carboxylic acid, methyl ester; TrxR, Thioredoxin reductase; DPI, Diphenyleneiodonium.

#### 2.4.1 Inhibition of GSH synthesis

GCL is the initial and rate-limiting enzyme in GSH production, and inhibitors of GCL may offer promising therapeutic strategies for cancers that exhibit heightened sensitivity to oxidative stress. The irreversible GCL inhibitor l-buthionine-sulfoximine (BSO) has shown good tolerability in Phase I clinical trials for neuroblastoma when combined with melphalan (NCT00005835, NCT00002730), though its standalone efficacy in inhibiting cancer growth remains limited [[67], [68], [69], [70]]. While BSO can potentiate the effects of platinum-based drugs like cisplatin and carboplatin [48,71], its clinical application is constrained by non-specific tissue toxicity and short half-life requiring prolonged infusions [72]. Notably, Liu's group demonstrated that combining BSO with the NADPH oxidase inhibitor DPI effectively kills cancer cells with HRAS G12V and KRAS mutations, suggesting a promising dual-targeting strategy for RAS-driven cancers [73].

NRF2 orchestrates a comprehensive antioxidant response by transcriptionally activating genes involved in GSH and thioredoxin metabolism, NADPH generation, quinone detoxification, and iron homeostasis [74]. While this program protects cells from oxidative damage, it also confers resistance to anticancer therapies by enabling tumor cells to counteract treatment-induced oxidative stress [75]. However, this adaptive response creates targetable metabolic vulnerabilities in cancer cells [76]. Most notably, it induces a dependence on glutaminolysis for glutamate production, a therapeutic vulnerability demonstrated in the KEAPSAKE trial evaluating the glutaminase inhibitor CB-839 in KEAP1/NRF2-mutant NSCLC (NCT04265534) [77]. The NRF2-KEAP1 axis precisely regulates GSH homeostasis: NRF2 activates γ-GCS and GR to boost GSH synthesis, while KEAP1 constitutively suppresses NRF2 to limit GSH production. Pharmacologically, Brusatol exploits this regulation by enhancing KEAP1-mediated NRF2 degradation, thereby depleting cellular GSH [78]. Although clinical trials like BeGIN (NCT03872427) have yet to yield significant breakthroughs, these mechanistic insights continue to guide therapeutic strategies targeting the NRF2 antioxidant network in cancer.

Studies have identified Auranofin and Stattic as effective GR inhibitors through distinct mechanisms. Auranofin inhibits thioredoxin reductase (TrxR), disrupting Trx system and forcing cells to compensate by overutilizing GSH for redox control [79]. Stattic, a STAT3 inhibitor, indirectly suppresses GSH production, leading to a reduction in GSH levels [80]. Activation of activating transcription factor 4 (ATF4) typically results in an increase in GSH levels, as ATF4 promotes GSH synthesis by upregulating the expression of γ-GCS [81]. The Integrated Stress Response Inhibitor (ISRIB) reduces GSH synthesis by inhibiting eIF2α phosphorylation, which subsequently decreases the translational activity of ATF4. This suppression of ATF4 activity leads to the downregulation of its target genes involved in GSH biosynthesis, thereby reducing GSH levels [82].

#### 2.4.2 Reduction of GSH precursors availability

The availability of GSH precursors can be decreased as an alternative method of lowering GSH levels. System Xc-, the glutamate/cysteine antiporter, composed of the subunits SLC7A11 (xCT) and SLC3A2 (CD98hc), functions as the main transporter for cystine uptake and glutamate efflux, with cystine being an essential precursor for GSH synthesis [83]. Sorafenib, an FDA-approved anticancer drug, inhibits the Xc-system, limiting cystine uptake, decreasing GSH synthesis, and increasing oxidative stress [84]. Importantly, by selectively inducing ferroptosis in tumor cells through this mechanism, Sorafenib achieves enhanced anticancer efficacy while sparing normal cells.

Similarly, the Xc-inhibitor sulfasalazine was investigated in a Phase I/II trial for treating progressive malignant glioma. However, the trial was halted early due to inadequate response and significant toxicity [84]. Erastin is another small molecule that binds to and inhibits SLC7A11 [85]. Research has demonstrated that Erastin potentiates the effects of tumor necrosis factor-related apoptosis-inducing ligand (TRAIL) in colon cancer cell lines [86]. Similar to BSO, Erastin inhibits cystine import, thereby inducing ferroptosis, and is hypothesized to contribute to the reduced radioresistance observed in lung cancer cells [87]. A Phase I clinical trial (NCT00528047) investigating the safety of the Erastin analogue PRLX 93936 in patients with advanced solid tumors was completed in 2012, followed by the initiation of a Phase I/II study (NCT01695590) in the same year [88].

Cells can acquire GSH precursors via the breakdown of extracellular GSH by GGT. Inhibitors of GGT, such as acivicin and 6-diazo-5-oxo-l-norleucine (DON), have been developed, but their use *in vivo* has been restricted due to toxicity issues [89].

#### 2.4.3 Promote the conversion of GSH to GSSG

GSSG is the oxidized form of GSH in redox reactions. The accumulation of GSSG is typically associated with a reduced antioxidant capacity and heightened oxidative stress. The GSH/GSSG ratio is commonly used to assess cellular redox status, with higher ratios indicating a reduced state and lower ratios reflecting increased oxidative stress. Elevated GSSG levels are closely linked to various diseases, with a particularly strong association in cancer cells. By promoting GSSG accumulation, it may enhance the efficacy of anticancer treatments, underscoring its significant biological relevance.

GPX4 reduces harmful L-OOH to their corresponding alcohols (L-OH) and converts GSH into GSSG [90]. Covalent inhibitors, such as (1S,3R)-RSL3 and ML162/ML210, irreversibly inhibit GPX4 activity by forming a covalent bond with its active site selenocysteine residue (Sec46) [91,92]. This binding leads to the buildup of lipid peroxides and induces ferroptosis. Additionally, PROteolysis TArgeting Chimeras (PROTAC) degraders exploit the unique characteristics of different cell types to precisely regulate GPX4 degradation [93].

#### 2.4.4 Promotion of GSH efflux

APR-246 is converted into methylene quinuclidinone (MQ), a Michael acceptor that interacts with thiol groups in both the antioxidant regulator TrxR and the antioxidant metabolite GSH, thereby inhibiting their activity. This mechanism of action not only reduces intracellular GSH levels but also indirectly promotes GSH efflux by increasing oxidative stress, further depleting intracellular GSH levels [94]. Studies have found that diphenyleneiodonium (DPI) promotes GSH efflux, with approximately 50 % of GSH exiting the cell within 2 h. This effect, which can be blocked by bromosulfophthalein (BSP), provides insight into DPI's pro-apoptotic actions and underscores the importance of regulating GSH homeostasis [95].

However, this area of research has only recently been explored. A deeper understanding of these pathways and the role of GSH within them will facilitate the creation of mechanism targeted GSH inhibitors, which can be paired with other therapeutic approaches to suppress tumor growth more effectively.

### 2.5 Summary

This part underscores the dual role of GSH in cancer, highlighting its pivotal functions in tumor initiation, progression, and therapy resistance while exploring its potential as a therapeutic target. GSH safeguards cells from oxidative stress during early carcinogenesis but also supports cancer cell survival by detoxifying carcinogens and maintaining redox homeostasis. The tumor microenvironment further exploits GSH. Therapeutic strategies targeting GSH metabolism include inhibiting synthesis, limiting precursors, promoting GSH oxidation, or enhancing efflux. Despite challenges like toxicity and limited clinical efficacy, these approaches reveal metabolic vulnerabilities and emphasize the need for combinatorial therapies. Here we provide a mechanistic framework for targeting GSH dynamics, linking redox biology to precision oncology and underscoring the therapeutic potential of disrupting GSH homeostasis while cautioning against nonspecific antioxidant use.

## 3 MicroRNA and their regulation of GSH metabolism

### 3.1 MicroRNA

MicroRNAs (miRNAs) are a group of non-coding RNAs typically about 22 nucleotides long. First discovered in the early 1990s, miRNAs have since been recognized as key controllers of numerous cellular functions, including differentiation, growth, and programmed cell death [96,97]. They modulate gene expression by interacting with the 3′ untranslated regions (3’ UTRs) of target mRNAs. This interaction generally leads to either the degradation or suppression of translation of the target mRNA [98].

The intricate, multi-step process of miRNA biogenesis is crucial for gene regulation. RNA polymerase II transcribes miRNA genes to produce hairpin-shaped primary miRNAs (pri-miRNAs). The microprocessor complex, consisting of the RNase III enzyme Drosha and its cofactor DGCR8, processes pri-miRNAs into precursor miRNAs in the nucleus. These precursors are then transported to the cytoplasm by Exportin-5, where Dicer, another RNase III enzyme, cleaves them into mature miRNA duplexes. These mature miRNAs guide target mRNA silencing via the RNA-induced silencing complex (RISC) [99,100].

Aberrant miRNA expression is strongly associated with numerous pathological conditions, particularly cancers. MiRNAs influence cellular responses to environmental changes, including the regulation of circadian rhythms [101,102]. Given their pivotal role in gene regulation, miRNAs hold significant potential for applications in cancer diagnosis and therapy [103].

### 3.2 MiRNA-mediated regulation of GSH metabolism

**Fig. 2:**
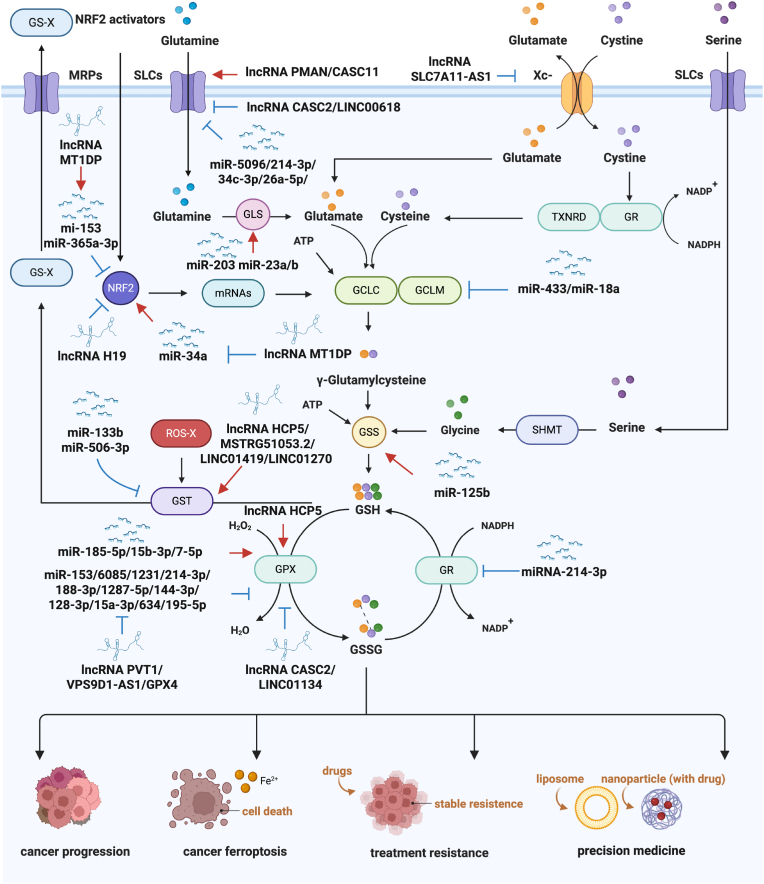
Description of various miRNAs and lncRNAs responsible for cancer progression and treatment resistance in various GSH metabolic pathways. miR-5096/214–3p/34c-3p/26a-5p and lncRNA CASC2/LINC00618 suppress solute carriers (SLCs)-mediated glutamine transport, significantly reducing GLS activity. Conversely, miR-203 and miR-23a/b positively regulate GLS expression to promote glutamate biosynthesis. lncRNA SLC7A11-AS1 inhibits xCT-mediated antiport of glutamate and cystine. lncRNA MT1DP blocks NRF2-mediated transcriptional activation of glutamate-cysteine ligase subunits GCLC and GCLM by upregulating miR-153 and miR-365a-3p while suppressing miR-34a. miR-433 and miR-18a directly downregulate GCLC/GCLM expression. miR-125b upregulates glutathione synthetase (GSS) expression. lncRNA HCP5 synergizes with miR-185–5p/156–3p/7–5p to enhance GPX-dependent peroxide decomposition, whereas lncRNA CASC2/LINC01134 and miR-153/6085/1231/214–3p counteract this process. The GST-mediated detoxification pathway, which utilizes GSH to eliminate peroxides and xenobiotics, is dynamically regulated by lncRNA HCP5/MSTRG5 1053.2 and miR-133b/506–3p. miR-214–3p impairs GR activity, disrupting GSH recycling. This regulatory network critically influences tumor progression, ferroptosis susceptibility, therapeutic resistance, and precision medicine strategies. Abbreviation: GLS, Glutaminase; GCLC, Glutamate-cysteine ligase catalytic subunit; GCLM, Glutamate-cysteine ligase regulatory subunit; GSS, Glutathione synthetase; GPX, Glutathione peroxidase; GST, Glutathione S-transferase; GR, Glutathione reductase; SLCs, Solute carriers; Xc-, Cystine/glutamate antiporter; xCT, cystine/glutamate exchange subtype, encoded by SLC7A11; NRF2, Nuclear factor erythroid 2-related factor 2; GSH, Glutathione; MRPs, Multidrug Resistance-associated Proteins; GS-X, Glutathione-conjugate; ROS-X, Reactive Oxygen Species-conjugate; SHMT, Serine Hydroxymethyltransferase; TXNRD, Thioredoxin Reductase.

The regulation of GSH by miRNAs is a complex process that involves multiple pathways. First, miRNAs modulate GSH synthesis by targeting key enzymes involved in its biosynthetic pathway (Fig. 2). For example, miR-433 and miR-18a have been shown to specifically target and downregulate the expression of GSH synthetase subunits GCLC and GCLM [104,105], directly affecting the rate of GSH synthesis [106,107]. MiR-433 directly suppresses GCL expression by targeting the 3′-untranslated regions (3′-UTRs) of both its catalytic subunit (GCLC) and regulatory subunit (GCLM), thereby reducing GSH synthesis independently of the Nrf2 pathway [104]. GSS facilitates the transformation of gamma-glutamylcysteine and glycine into GSH, utilizing ATP as a cofactor [108]. Studies have indicated that miR-125b regulates GSS expression, thereby increasing the cells' capacity to produce GSH [109]. The regulation of NRF2 by miR-34a, miR-365a-3p and miR-153 impacts GSH metabolism [[110], [111], [112]]. Additionally, miR-23a and miR-24b repression resulted in the increased expression of GLS [113]. MiR-203 was also reported to regulate GLS, indirectly increases GSH abundance via reduced glutamate availability [114].

In addition to regulating GSH metabolism, miRNAs can regulate the expression of genes involved in ROS clearance, thereby altering GSH levels. Key enzymes in the detoxification of ROS include GPX and superoxide dismutase (SOD). SOD serves as the primary defense against ROS by converting O_2_•^-^ into H_2_O_2_, thereby mitigating oxidative damage [115]. GPX4, in contrast, plays a key role in preventing lipid peroxidation and ferroptosis by using GSH to reduce both H_2_O_2_ and lipid peroxides (L-OOH), offering protection against oxidative stress. Studies have shown that miR-21 and miR-153 target antioxidant enzymes such as SOD and GPX, thereby influencing the redox status of GSH [112,116]. Moreover, the regulation of these enzymes by miRNAs directly impacts the role of GSH in safeguarding cells from oxidative damage. The efficiency of ROS scavenging is closely linked to GSH levels, as GPX4 relies on GSH to function. When ROS levels increase or when SOD and GPX are inactivated, GSH is rapidly consumed to neutralize the excess ROS. Conversely, if GPX4 activity is reduced, GSH may accumulate, but the overall antioxidant capacity of the cell is compromised, leading to increased oxidative stress and an elevated risk of ferroptosis [117].

### 3.3 MiRNA-GSH regulation in cancer progression

The critical role of miRNAs underscores their potential as therapeutic targets in medical interventions for oxidative stress-related disorders, including cancer. The interaction between miRNAs and GSH during tumor progression significantly influences tumor cell behavior (Table 2).

**Table 2:** miRNA-Mediated GSH Regulation in Cancer.

miRNA	Target	Redox Enzymes Affected	GSH Level	Oxidative Stress	Cancer Type	Regulation	Effect	Ref
miR-433	GCLC, GCLM	GCLC, GCLM	↓	↑	Various cancers	Inhibits GSH synthesis	Reduces antioxidant capacity	[104]
miR-18a	GCLC	GCLC	↓	↑	Liver cancer	MYC-induced suppression of GSH synthesis	Enhances oxidative stress sensitivity	[105]
miR-125b	PCTP, LIPA and GSS	GSS	↑	↓	Chronic lymphocytic leukemia (CLL)	Regulates transcripts encoding enzymes and factors in metabolism	Promotes tumor survival and therapy resistance	[109]
miR-34a	NRF2	–	↑	↓	Colorectal cancer	Enhances antioxidant defenses	Promotes tumor growth and chemotherapy resistance	[110]
miR-365a-3p	NRF2	HO-1, NQO1	↓	↑	NSCLC	Disrupts antioxidant defenses	Promotes chemoresistance	[111]
miR-153	NRF2 GPX1	GPx1	↓	↑	Glioma	Disrupts antioxidant defenses	Promotes stemness and radioresistance	[112]
miR-23a/b	Mitochondrial GLS	GLS	↑ (implied by reduced glutamate availability)	↓	B-cell lymphoma, Prostate cancer	Suppresses glutamine catabolism	Promotes proliferation	[113]
miR-203	GLS	GLS	↑ (implied by reduced glutamate availability)	↓	MM	Suppresses glutamine metabolism	Promotes chemosensitivity	[114]
miR-21	SOD3, TNF-α, SOD2	SOD3, SOD2	–	↑O_2_•^-^ ↓ H_2_O_2_ conversion	Pan-cancer	Disrupts ROS metabolism	Promotes tumorigenesis	[116]
miR-5096	SLC7A11	–	↓	↑	Breast cancer	Reduces cystine uptake, lowering GSH	Promotes ferroptosis, inhibits tumor growth	[119]
miR-506–3p	GSTP1	GST	↓	↑	CRC	Suppresses GSTP1, reducing GSH conjugation	Increases chemotherapy sensitivity	[120]
miR-214–3p	ATF4 GR SLC3A2	GR COX2, ACSL4, PTGS2, GPX4, NOX1	↓	↑	HCC	Inhibits ATF4-mediated antioxidant response, downregulates SLC3A2	Promotes ferroptosis, inhibits tumor growth	[123,129,130]
miR-103a-2-5p	LILRB3	Nrf2, HO-1	↓	↑	AML	Downregulates LILRB3	Inhibits AML cell proliferation, reduces CD8^+^ T cell apoptosis, suppresses tumor growth	[126]
miR-185–5p	GPX1	GPX1	↑ (implied by GPX1 ↓)	↓	AML	Downregulates GPX1	Blocks AML progression	[127]
miR-6085	AIFM2, GPX4	GPX4	↓ (implied by ↑ ferroptosis)	↑	HCC	Directly targets AIFM2 & GPX4	Promotes ferroptosis, inhibits HCC progression	[128]
miR-1231	GPX4	GPX4	↓ (implied by ↑ ferroptosis)	↑	PTC	Directly targets GPX4, inducing ferroptosis	Inhibits cancer progression	[131]
miR-188–3p	GPX4	GPX4	↓ (implied by ↑ ferroptosis)	↑	OS	Directly targets GPX4, inducing ferroptosis	Inhibits cancer progression	[132]
miR-1287–5p	GPX4	GPX4	↓ (implied by ↑ ferroptosis)	↑	OS	Downregulates GPX4, inducing ferroptosis	Suppresses tumor progression	[133]
miR-26b-5p	MAT2A	–	↓	↑	OS	Directly targets MAT2A, activates STAT3/SLC7A11signaling	Suppresses tumor proliferation/invasion	[134]
miR-144–3p	ZEB1	GPX4	↓ (implied by ↑ ferroptosis)	↑	OS	Directly targets ZEB1, inducing ferroptosis	Suppresses tumor proliferation/migration/invasion	[135]
miR-128–3p	SP1/CD98hc	GPX4	↓ (implied by ↑ ferroptosis)	↑	Breast Cancer	Negatively regulates GPX4, inducing ferroptosis	Suppresses tumor migration/invasion/stemness	[136]
miR-15a-3p	GPX4	GPX4	↓ (implied by ↑ ferroptosis)	↑	CRC	Negatively regulates GPX4	Induces ferroptosis	[137]
miR-634	GPX4	GPX4	↓ (implied by ↑ ferroptosis)	↑	PC	Negatively regulates GPX4	Induces ferroptosis	[138]
miR-34c-3p	SLC7A11	GPX4	↓ (implied by ↑ ferroptosis)	↑	OSCC	Negatively regulates GPX4	Induces ferroptosis	[216]
miR-26a-5p	SLC7A11	GPX4	↓ (implied by ↑ ferroptosis)	↑	OSCC	Negatively regulates GPX4	Induces ferroptosis	[140]
miR-122–5p	CS	–	↓	↑	NPC	Negatively regulates CS	Induces ferroptosis	[141]
miR-133b	GSTP1	GST	↓	–	NSCLC	Negatively regulates GSTP1	Inhibits cisplatin resistance and tumor progression	[143]
miR-497	TKT	pentose phosphate pathway	↓	↑	Cervical cancer	Negatively regulates TKT	Inhibits cisplatin resistance	[144]
miR-195–5p	PLAG1, PVT1	GPX4	↓ (implied by ↑ ferroptosis)	↑	HCC	Negatively regulates PLAG1/PVT1	Induces ferroptosis	[147]
miR-15b-3p	KLF2	GPX4	↑ (implied by ↑ ferroptosis)	↓	PCa	Negatively regulates KLF2	Inhibits ferroptosis, promotes BIC resistance	[148]
miR-7-5p	ACVRL1	GPX2	↑ (implied by GPX2 ↑)	↓	CRC	Downregulated by the Wnt/β-catenin/TCF-1-KCNQ1OT1 axis	Inhibition of miR-7-5p promotes resistance to mTKIs	[150]

GCLC, Glutamate-cysteine ligase catalytic subunit; GCLM, Glutamate-cysteine ligase modifier subunit; GSS, Glutathione synthetase; NRF2, Nuclear factor erythroid 2-related factor 2; HO-1, Heme oxygenase-1; NQO1, NAD(P)H quinone dehydrogenase 1; GPX1, Glutathione peroxidase 1; GLS, Glutaminase; SOD3, Superoxide dismutase 3; SOD2, Superoxide dismutase 2; TNF-α, Tumor necrosis factor-alpha; SLC7A11, Solute carrier family 7 member 11 (cystine/glutamate transporter, xCT); GST, Glutathione S-transferases; GSTP1, Glutathione S-transferase Pi 1; ATF4, Activating transcription factor 4; GR, Glutathione reductase; SLC3A2, Solute carrier family 3 member 2 (CD98 heavy chain); COX2, Cyclooxygenase-2; ACSL4, Acyl-CoA synthetase long-chain family member 4; PTGS2, Prostaglandin-endoperoxide synthase 2; NOX1, NADPH oxidase 1; LILRB3, Leukocyte immunoglobulin-like receptor subfamily B member 3; AIFM2, Apoptosis-inducing factor mitochondria-associated 2; PTC, Papillary thyroid carcinoma; OS, Osteosarcoma; MAT2A, Methionine adenosyltransferase 2A; STAT3, Signal transducer and activator of transcription 3; ZEB1, Zinc finger E-box binding homeobox 1; SP1, Specificity protein 1; CD98hc, CD98 heavy chain (SLC3A2); PC, Pancreatic cancer; OSCC, Oral squamous cell carcinoma; CS, Citrate synthase; NPC, Nasopharyngeal carcinoma; TKT, Transketolase; PLAG1, Pleomorphic adenoma gene 1; PVT1, Plasmacytoma variant translocation 1; KLF2, Kruppel-like factor 2; PCa, Prostate cancer; BIC, Bicalutamide; CRC, Colorectal cancer; mTKIs, Multitarget tyrosine kinase inhibitors; ROS, Reactive oxygen species; GSH, Glutathione; NSCLC, Non-small cell lung cancer; HCC, Hepatocellular carcinoma; MM, Multiple myeloma; AML, Acute myeloid leukemia; CLL, Chronic lymphocytic leukemia.

MYC is a proto-oncogene encoding a transcription factor that plays a crucial role in cell growth, proliferation, and metabolism [118]. Anderton et al. reported that MYC activation in liver cancer enhanced sensitivity to oxidative stress and promoted tumor growth by inhibiting GCL activity and GSH synthesis [105]. This effect is attributed to MYC-induced microRNA miR-18a. MiR-5096 inhibits tumor growth in breast cancer cells by targeting SLC7A11, reducing GSH levels and increasing ROS and lipid ROS levels [119]. Additionally, miR-506–3p suppresses cell growth and increases cell death in colorectal cancer (CRC) cells by downregulating GSTP1 [120]. GSTP1 encodes GST enzymes that detoxify CRC cells from harmful compounds by utilizing GSH [121].

Overexpression of miR-214 is linked to poor cancer prognosis, as it induces oxidative stress by downregulating the expression of GR, a factor associated with the progression of various cancers [[122], [123], [124], [125]]. Another study showed that CLPs-miR-103a-2-5p suppressed the growth and led to programmed cell death in acute myeloid leukemia (AML) cells by specifically interacting with LILRB3 and modulating the Nrf2/HO-1 pathway [126]. Additionally, miR-185–5p inhibited the expression of GPX1, which reduced AML cell proliferation and invasion while promoting cell differentiation and death [127]. The expression of GSS inversely correlates with miRNA-125b levels, demonstrating tumor-suppressive effects in chronic lymphocytic leukemia patients [109].

### 3.4 MiRNA-GSH regulation in cancer ferroptosis

The miRNA-GSH axis is critical in regulating ferroptosis in tumors. By modulating GSH, a key antioxidant, miRNAs influence ferroptosis sensitivity, affecting tumor survival and therapy response (Table 2).

A study found that the release of miR-6805 from its target genes, apoptosis-inducing factor mitochondria-associated 2 (AIFM2) and GPX4, inhibited ferroptosis and promoted tumor development in hepatocellular carcinoma (HCC) [128]. Exosomal miR-142–3p, released by HBV-related HCC cells, induces ferroptosis in M1 macrophages by targeting SLC3A2, which may contribute to the progression of HCC [129]. Ferroptosis in HCC cells were driven by the upregulation of miR-214–3p, which suppressed the expression of ATF4, reduced intracellular GSH levels, and activated the antioxidant enzyme GPX4 [130]. Downregulation of miR-1231 and miR-188–3p increased GPX4 expression and intracellular GSH levels, thereby suppressing ferroptosis and promoting progression in papillary thyroid cancer and osteosarcoma, respectively [131,132]. In contrast, miR-1287–5p inhibited tumor development in osteosarcoma cells by downregulating GPX4 and inducing ferroptosis [133]. Previous studies have shown that miR-26b-5p targets methionine adenosyltransferase 2A (MAT2A), leading to reduced GSH levels and promoting ferroptosis by blocking the STAT3/SLC7A11 pathway in osteosarcoma [134]. MiR-144–3p reduces GSH levels by targeting and downregulating ZEB1, which in turn decreases the expression of genes like SLC7A11, reducing cystine availability for GSH synthesis and promoting ferroptosis. This action inhibits osteosarcoma cell proliferation, migration, and invasion [135].

Another study examined how Empagliflozin could treat breast cancer by activating miR-128-3p-dependent pathways and inhibiting CD98hc, leading to reduced GSH levels and triggering ferroptosis in anti-apoptotic cells [136]. In CRC, miR-15a-3p decreased tumor development by reducing GPX4 expression, promoting ferroptosis [137]. As a molecular sponge, circ_WASF2 promotes pancreatic cancer progression by regulating the miR-634/GPX4 signaling pathway [138]. Additionally, upregulation of miR-34c-3p results in downregulation of SLC7A11 expression, decreased GSH levels, reduced GPX4 activity, and increased ROS levels, thereby promoting ferroptosis and inhibiting cell growth in oral squamous cell carcinoma (OSCC) [139]. Furthermore, hsa-miR-26a-5p increased the susceptibility of OSCC cells to ferroptosis by suppressing SLC7A11 [140]. In nasopharyngeal cancer, miR-122–5p modulates citrate synthase (CS) and GSH levels, promotes ferroptosis, and may influence tumor growth and treatment response [141].

### 3.5 MiRNA-GSH regulation in treatment resistance

The interaction between miRNAs and GSH synthesis enzymes is particularly critical in the context of developing resistance to chemotherapy drugs [107,142]. For instance, miR-133b, which downregulates GSTP1, has been shown to reverse cisplatin resistance, increase cell susceptibility to cisplatin, and induce cell death in NSCLC [143]. Additionally, MiR-497 improves the susceptibility of cervical cancer cells to cisplatin by inhibiting transketolase (TKT), lowering GSH levels, and increasing ROS production [144].

As mentioned previously, SLC7A11 is a glutamate-cysteine transporter involved in GSH production. Pleiomorphic Adenoma Gene 1 (PLAG1) is a transcription factor that plays a role in diverse biological processes such as tumor progression, cell redox homeostasis, and ferroptosis, emerging as a potential target for cancer research and therapy [145,146]. A study by Li et al. reported that miR-195–5p knockdown in HCC led to increased GSH levels by upregulating PLAG1 and GPX4, thereby enhancing resistance of HCC cells to sorafenib [147].

MiR-15b-3p reduces cell death by targeting KLF2, which decreases the susceptibility of prostate cancer to bicalutamide. Upregulation of miR-15b-3p may alter GSH levels and antioxidant enzyme activity [148]. These changes collectively contribute to the development of drug resistance in prostate cancer, potentially promoting tumor progression and leading to poor prognosis. Activin A receptor-like type 1 (ACVRL1) is a multifunctional signaling molecule that serves an important function in angiogenesis and cancer development [149]. Recent studies have shown that ACVRL1 is crucial in resistance to multi-target tyrosine kinase inhibitors. Suppressing miR-7-5p increases ACVRL1 expression, while stabilizing GPX2 reduces intracellular H_2_O_2_ levels, thereby boosting resistance to Regorafenib and Sorafenib in CRC cells [150].

MiR-125b is downregulated in chronic lymphocytic leukemia (CLL), leading to increased GSS expression and elevated GSH levels, which contribute to resistance to radiotherapy and chemotherapy [109]. These studies emphasize the critical role of the interaction between miRNAs and GSH in tumor cell responses to chemotherapy and radiotherapy, offering new targets and potential treatment strategies for cancer therapy (Table 2).

### 3.6 GSH-regulation-based miRNA-targeted applications in cancer

Innovative approaches such as nanoprobes, nanocomposites, and drug delivery systems targeting specific miRNAs based on GSH regulation offer significant potential for tumor detection, inhibition of tumor progression, and enhancement of anticancer treatment efficacy [[151], [152], [153]] (Table 3).

**Table 3:** Novel Applications of miRNA Based on GSH Regulation in Cancer.

Application	miRNA	Delivery Systems	Mechanism of Release	Mechanism of Action	Cancer	Ref
Imaging	miR-155	MnO_2_ nanosheets	GSH reduces MnO_2_ nanosheets to Mn^2+^ inside cells and releases probes	Activated DNAzyme cleavage hybridizes with miRNA-155 to disrupt FRET, generating detectable signals	Cervical cancer liver cancer	[155]
Imaging	–	core-shell nanomaterial (MSNs@MnO_2_)	Intracellular GSH reduces MnO_2_ shell and decomposes nanosheets	GSH-responsive MnO_2_ degradation mediates dual-signal fluorescence recovery (R6G + miRNA beacons) for co-detection of redox/miRNA	various cancers	[156]
Imaging	–	MnPFEDz nanoprobe	Intracellular GSH reduces MnO_2_ nanosheets and generates Mn^2+^ cofactors	miRNA initiates Mn^2+^-powered DNAzyme/EDC cascades for autonomous signal amplification	various cancers	[157]
Diagnosis/Therapy	miR-21	CGT probe (MnO_2_ + DNA)	GSH degrades the CGT probe, releasing Mn^2+^ and DNA	Mn^2+^ enables CDT via Fenton-like reactions, while enzyme-like activities drive ST, synergizing therapy and miRNA detection	various cancers	[158]
Diagnosis	miR-103a	Cu superparticles	–	Cu superparticles generate strong AIE-ECL signals upon miRNA-103a binding via CHA	TNBC	[159]
Therapy	miR-34a	Nanocomplexes	Intracellular GSH cleaves the disulfide bonds in S-Arg_4_, triggering nanocomplex dissociation and simultaneous release of miR-34a and ICG.	miR-34a exerts tumor-suppressive effects, while ICG's restored NIRF enables real-time monitoring of miRNA replacement efficacy, correlating with therapeutic outcomes	HCC, breast cancer	[160]
Therapy	miR-448	miDAC@PDA	Intracellular GSH and laser-induced hyperthermia trigger dissociation of the PDA shell, releasing miRNA and DOX/ATP/Cu^2+^ from the miDAC core.	miR-448 silences IDO1 to reverse immunosuppression, while Cu^2+^-mediated GSH depletion and ATP-enhanced DOX amplify ICD, synergizing with photothermal therapy for tumor regression	various cancers	[161]
Therapy	miR-133a	HPAA polymer	Intracellular GSH triggers the degradation of the cationic HPAA polymer, releasing miRNA-133a-3p from the nanocomplex via breakdown of electrostatic interactions	miRNA-133a-3p exerts tumor-suppressive effects, while PSMA-targeted aptamer ensures selective uptake in prostate cancer cells, inhibiting bone metastasis progression	Prostate cancer	[162]
Therapy	miR-155	FTP polyantioxidant	Intracellular GSH cleaves the redox-responsive linker in FTP, triggering the release of both TEMPOL antioxidant and anti-miR-155	TEMPOL scavenges ROS while anti-miR-155 inhibits oncogenic miR-155, jointly suppressing the NF-κB pathway to block cancer metastasis	Breast cancer	[163]
Therapy	miR-155	ROS/GSH-sensitive NPs	The ROS/GSH dual-responsive CUR/miR155@DssD-Hb NPs degrade in tumor and release both curcumin (CUR) and miR-155	miR-155 enhances DC maturation and M1 macrophage polarization, while CUR counteracts immunosuppression by reducing Tregs, MDSCs, and M2 TAMs, synergistically promoting cytotoxic T-cell activation and long-term anti-tumor immunity	Melanoma, TNBC	[164]
Therapy	miR-122	Graphene/InP nanocomposites (GPMQNs)	Intracellular GSH triggers the controlled release of miR-122 from GPMQNs	miR-122 induces apoptosis in liver cancer cells, while InP@ZnS quantum dots enable targeted near-infrared imaging for tumor visualization	Liver cancer	[165]
Therapy	miR-124–5p	AuNP aggregates cross-linked by cystamine	Elevated GSH levels cleave nanocarrier disulfide bonds, releasing miRNA	The release of miRNA inhibits tumor gene expression, while the photothermal effect under NIR irradiation eradicates tumor cells	Cervical cancer, colon adenocarcinoma	[152]

GSH, Glutathione; FRET, Fluorescence Resonance Energy Transfer; MSNs@MnO_2_, Mesoporous Silica Nanoparticles@Manganese Dioxide; R6G, Rhodamine 6G; MnPFEDz, (Context-dependent, typically a manganese-based complex; exact full name may vary); EDC, 1-Ethyl-3-(3-dimethylaminopropyl)carbodiimide; CGT, Cancer-targeted Glutathione-gated Theranostic probe; ST, Starvation Therapy; AIE-ECL, Aggregation-Induced Electrochemiluminescence; TNBC, Triple-Negative Breast Cancer; HCC, Hepatocellular Carcinoma; ICG, Indocyanine Green; miDAC@PDA, miRNA-loaded Drug-Amplified Coordination polymer@Polydopamine; PDA, Polydopamine; ATP, Adenosine Triphosphate; DOX, Doxorubicin; ICD, Immunogenic Cell Death; HPAA, Hyperbranched Poly(amido amine); PSMA, Prostate-Specific Membrane Antigen; FTP, Fluorinated-TEMPOL-Polyethyleneimine (poly-antioxidant); TEMPOL, 4-Hydroxy-2,2,6,6-tetramethylpiperidin-1-oxyl; ROS, Reactive Oxygen Species; NPs, Nanoparticles; CUR/miR155@DssD-Hb, Curcumin/miR-155@Disulfide-crosslinked Hemoglobin-based Nanoparticles; DC, Dendritic Cells; CUR, Curcumin; MDSC, Myeloid-Derived Suppressor Cells; TAM, Tumor-Associated Macrophages; GPMQNs, Graphene-P-glycoprotein/miR-122-InP@ZnS Quantum Dots Nanocomposites; InP@ZnS, Indium Phosphide/Zinc Sulfide; NIR, Near-Infrared.

Nanopore technologies for cancer biomarker detection are continually advancing [154]. Ratiometric fluorescent probes incorporating active DNase in manganese oxide (MnO_2_) nanosheets have been proposed for detecting and imaging miRNAs in living cells [155]. Intracellular GSH reduces MnO_2_ to Mn^2+^, releasing probes and activating DNAzyme cleavage, which hybridizes with miRNA-155 to disrupt FRET (fluorescence resonance energy transfer), generating a detectable signal. Additionally, a novel core-shell “loading-type” nanomaterial (MSNs@MnO_2_) was designed to allow simultaneous imaging of GSH and microRNA through GSH-triggered release and FRET imaging [156]. A catalyst-DNAzyme circuit (MnPFEDz) was used to create a self-powered nanoprobe that reports entropy, providing high-sensitivity miRNA imaging in live cells [157].

Su et al. developed a GSH-gated probe (CGT probe) that utilizes intracellular GSH overexpression to activate probe unlocking and release functional DNA, enabling effective cancer therapy and miRNA imaging [158]. An enhanced aggregation induction strategy based on copper superparticles was developed to detect miRNA-103a in triple-negative breast cancer (TNBC) using GSH-terminated copper clusters as precursors [159].

A study demonstrated a novel strategy to deliver miR-34a using nanocomplexes, which increased intracellular GPX and GSH levels while decreasing ROS levels, ultimately inhibiting tumor development through apoptosis induction by targeting proteins such as BCL-2 [160]. Researchers developed a laser/GSH-activated miRNA coordination polymer nanocomplex (miDAC@PDA) for combination immunotherapy, photothermal therapy, and chemotherapy [161]. Ye et al. created a PSMA-targeted, reduced-cleavable hyperbranched polyamidoamine (HPAA) gene delivery system for bone metastases of prostate cancer. The miRNA-133a-3p within this system demonstrated potent anti-tumor effects observed both *in vivo* and *in vitro*. When GSH levels are high, this GSH-responsive system releases medication into the tumor microenvironment, improving treatment efficacy and specificity [162]. Additionally, a novel polyantioxidant (FTP) was developed to target miR-155, enhancing treatment for metastatic breast cancer through ROS scavenging and miR-155 inhibition [163]. Another study demonstrated that ROS/GSH-sensitive nanoparticles efficiently release loaded miR-155 and curcumin in a high GSH environment, enhancing cancer cell death by modulating H_2_O_2_ levels and miR-155 expression [164]. Zeng et al. studied the targeting and induction of apoptosis in resistant HCC cells using miR-122-loaded graphene/indium phosphide nanocomposites. Upregulation of miR-122 effectively overcame Adriamycin resistance in liver cancer cells, promoting apoptosis. However, the release of miR-122 was significantly influenced by the GSH level [165]. Further advancing combinatorial therapies, a GSH-responsive gold nanoparticle (AuNP) aggregate system was engineered to co-deliver miR-124–5p and photothermal therapy. Upon cytoplasmic GSH-triggered release of miR-124–5p, the AuNP aggregates maintained structural stability via thiol-gold bond reformation, enabling sustained photothermal ablation. This dual-modality approach exhibited 2–3 times greater cytotoxicity than single therapies, highlighting its potential for synergistic tumor treatment [152].

### 3.7 Summary

MiRNAs modulate GSH metabolism by targeting key enzymes such as GCLC, GCLM, GSS, and GPX4, as well as transcription factors like NRF2, thereby influencing GSH synthesis, redox balance, and ferroptosis sensitivity. In cancer progression, miRNAs alter GSH levels, impacting tumor growth and oxidative stress responses. Ferroptosis regulation by miRNAs modulating GPX4 and SLC7A11 to either suppress or promote ferroptosis in hepatocellular carcinoma, osteosarcoma, and other cancers. Additionally, miRNAs influence therapy resistance, making them ideal targets for cancer therapy. The primary challenge in current miRNA therapeutics lies in achieving target specificity, as individual miRNAs can regulate multiple genes, necessitating urgent resolution of off-target effects. Studies have shown that nonspecific gene silencing may lead to neurotoxicity and immunotoxicity, which in turn reduces therapeutic efficacy [166]. This calls for in-depth studies to clarify the multiple target gene networks of miRNAs and systematically evaluate their off-target effects.

GSH-regulated miRNA delivery systems, including GSH-responsive nanoprobes and nanocomplexes, demonstrate promise for precise tumor imaging and targeted therapy. Innovations such as laser/GSH-activated miRNA nanocomplexes and polyantioxidant-based miRNA inhibitors highlight the potential for combinatorial strategies integrating photothermal therapy, immunotherapy, and redox modulation. Another key challenge remains in clinical translation. It is necessary to develop an ideal delivery system that combines high efficacy, low toxicity and cost-effectiveness. The delivery system needs to be optimized to ensure that the exogenous miRNA can be delivered efficiently and integrated with the genome to perform its function. miRNA therapies can also be used in combination with existing chemotherapies or radiotherapies, and the chemo-sensitizing properties of some miRNAs will provide additional benefits to the combination therapy. Preclinical and clinical studies are currently underway, and in the future, it is expected that customized miRNA sequences will be developed based on individual genomes, enabling truly personalized cancer therapy.

## 4 LncRNA and their regulation of GSH metabolism

### 4.1 LncRNA

Long non-coding RNAs (lncRNAs) are RNA molecules exceeding 200 nucleotides in length that do not encode proteins [167]. These molecules exhibit a wide range of molecular features, including structural variability, tissue-specific expression, and disease-associated expression patterns. Compared to other RNA types, lncRNAs generally display lower expression levels and greater tissue specificity [168]. Based on their genomic location and functional roles, lncRNAs can be categorized into various groups, including natural antisense transcripts, long intergenic non-coding RNAs (lincRNAs) and intragenic lncRNAs [169]. LncRNAs play pivotal roles in regulating gene expression, epigenetic modifications, cellular differentiation, and various other cellular processes, with some interacting directly with chromatin remodeling complexes to modulate transcriptional activity [170]. These molecules often engage in complex interactions with proteins, mRNAs, and other non-coding RNAs, establishing intricate regulatory networks [[171], [172], [173]]. Moreover, many lncRNAs are closely linked to the development and progression of various diseases, including cancer, where they can serve as potential biomarkers or therapeutic targets due to their disease-specific expression profiles [174,175].

### 4.2 LncRNA-mediated regulation of GSH metabolism

The involvement of lncRNAs in GSH metabolism has become increasingly recognized as a critical factor in regulating oxidative stress responses, cellular proliferation, and apoptosis, particularly in the context of cancer (Fig. 2).

The regulation of GSH by lncRNAs involves multiple mechanisms, including the modulation of genes responsible for GSH synthesis, recycling, or degradation. LncRNA H19 has been identified as a key regulator of NRF2-targeted genes, such as NQO1, GR, G6PD, GCLC, GCLM, and GSTP1 [176,177]. Knockdown of H19 results in reduced GSH levels, further emphasizing its pivotal role in GSH regulation. LncRNAs can also contribute to the modulation of mitochondrial GSH levels. For instance, LINC00493 promotes mitochondrial glutathione import by interacting with SLC25A11, a key transporter involved in mitochondrial GSH uptake [178].

LncRNAs also play a significant role in regulating various enzymes that modulate GSH levels. In liver cancer, the lncRNA PVT1 has been shown to regulate GPX4 expression through activating STAT3, thereby affecting redox homeostasis [179]. Other lncRNAs regulate enzymes such as MGST1, MGST3, SOD, and TALDO1, can potentially impact GSH metabolism, thereby influencing GSH levels and the cellular oxidative stress response [[180], [181], [182], [183]]. Linc-ROR in HCC modulates GSH levels and active oxidative stress [182]. Although the precise mechanism linking Linc-ROR to GSH has not been fully elucidated, its involvement in arsenic trioxide (ATO) resistance suggests a regulatory impact on the oxidative stress response, potentially through the modulation of GSH metabolism.

The aforementioned examples demonstrate the diverse mechanisms through which lncRNAs regulate GSH metabolism, underscoring the complexity of cancer biology. By modulating key enzymes and transporters involved in GSH synthesis, recycling, and utilization, lncRNAs not only influence the cellular antioxidant defense but also play critical roles in cancer progression, therapy resistance, and the regulation of cell death pathways (Table 4).

**Table 4:** LncRNA-mediated regulation of glutathione metabolic pathways in cancer.

lncRNA	Target	Redox Enzymes Affected	GSH Level	Oxidative Stress	Cancer Type	Regulation	Effect	Ref
H19	NRF2	NQO1, GR, G6PD, GCLC, GCLM, GSTP1	↓ (upon H19 knockdown)	↑ (upon H19 knockdown)	Multiple cancers	Transcriptional regulation of NRF2 targets	Promotes antioxidant defense, cancer progression	[176]
LINC00493	AGK, SLC25A11	–	Maintain mitoGSH import	–	ccRCC	Binds AGK and SLC25A11 via N-terminus	Inhibits cancer metastasis.	[178]
PVT1	STAT3/GPX4 axis; miR-214–3p/GPX4 axis	GPX4	↓ (when PVT1 inhibited)	↑ (when PVT1 inhibited)	OS; HCC	Activates STAT3 signaling and upregulates GPX4; Sponges miR-214–3p and upregulates GPX4	Inhibits ferroptosis, promotes proliferation/metastasis	[179,187]
XLOC_009190	MGST1	MGST1	Potentially modulates (via MGST1 activity)	–	Lung adenocarcinoma	*Cis*-regulation of MGST1; *trans*-regulation of GPCRs/potassium channels	Participate in cellular protection via GSH conjugation	[180]
MSTRG51053.2	miR-432–5p/MGST3	MGST3, MGST1, GST-ω1	↑ (implied by ↑ MGST3/GST activity)	↓ (implied by ↑ detoxification)	NSCLC	ceRNA to sponge miR-432–5p, upregulating MGST3	Promotes cisplatin resistance	[181]
LncRNA ROR	p53	SOD	↓ (upon ATO treatment)	↑	HCC	Induced by ATO via oxidative stress, sponges/represses p53 expression	Confers ATO resistance	[182]
lnc-AP (encodes pep-AP)	TALDO1	TALDO1	↓	↑	CRC	pep-AP binds and suppresses TALDO1, attenuates PPP, inhibits NADPH/GSH	Sensitizes CRC to oxaliplatin	[183]
NUTM2A-AS1	miR-613/VEGFA	SOD	↑	↓	GC	Sponges miR-613, upregulates VEGFA, modulates redox balance	Confers matrine resistance	[184]
VPS9D1-AS1	miR-491–5p/miR-214–3p/GPX1	GPX1	↑ (implied by ↓ GPX1-mediated GSH consumption)	↓	ALL	Acts as a ceRNA to sponge miR-491–5p/miR-214–3p and upregulates GPX1	Promotes ALL progression	[186]
NEAT1	miR-362–3p/MIOX	MIOX	↓	↑	HCC	NEAT1 sponges miR-362–3p and upregulates MIOX	Promotes ferroptosis, sensitizes HCC to erastin/RSL3	[188]
PMAN	ELAVL1/SLC7A11	–	↑	↓	GC	PMAN binds ELAVL1, stabilizes SLC7A11 mRNA	Inhibits ferroptosis, Promoting PM survival under hypoxia	[189]
CASC2	FMR1/SOCS2/SLC7A11	GPX4	↓	↑	GC	CASC2 stabilizes SOCS2 mRNA and ubiquitinates SLC7A11	Promotes ferroptosis	[190]
HCP5	GPX4	GPX4	↑ (implied by GPX4-mediated GSH recycling)	↓	TNBC	Encoded protein HCP5-132aa upregulates GPX4 expression	Promotes TNBC progression	[191]
LINC00618	LSH/SLC7A11/BAX	–	↓	↑	Leukemia	Downregulates LSH and SLC7A11 transcription; upregulates BAX/caspase-3	Dual pro-death role	[192]
GSEC	miR-101–3p/CISD1/ATP5MC3/PGD	–	↑ (implied by GSH metabolism)	–	LUAD	Sponges miR-101–3p and upregulates CISD1/ATP5MC3/PGD; activates ferroptosis/glutathione pathways	Promotes LUAD progression	[193]
CASC11	SLC7A11	–	↑	↓	HCC	Binds/stabilizes SLC7A11 mRNA, upregulates SLC7A11 protein expression	Inhibits sorafenib-induced ferroptosis	[194]
SLC7A11-AS1	xCT	GCLM	↓	↑	GC	Directly suppressed xCT expression, low SLC7A11-AS1 activated expression of GCLM	Sensitizes GC to cisplatin	[195]
GDIL	CHAC1	CHAC1	↑	↓	CRC, ovarian cancer	re-localizes XRN2 protein to cytoplasm, XRN2 further degrades CHAC1 mRNA	Induces platinum resistance	[196]
MT1DP	miR-365a-3p/NRF2	NRF2-regulated enzymes (e.g., HO-1, NQO1)	↓	↑	NSCLC	Stabilizes miR-365a-3p and downregulates NRF2	Promotes ferroptosis	[111]
LINC01419	GSTP1 promoter	GSTP1	↑ (implied by GSTP1 suppression)	–	ESCC	Binds GSTP1 promoter, recruits DNMTs, hypermethylates GSTP1, downregulates GSTP1 expression	Promotes ESCC progression	[197]
LINC01270	GSTP1 promoter	GSTP1	↑ (implied by GSTP1 suppression)	–	EC	Recruits DNMT1/DNMT3A/DNMT3B, hypermethylates GSTP1 promoter, downregulates GSTP1 expression	Promotes EC progression	[198]
ITGB2-AS1	FOSL2/NAMPT/p53	GPX4	↑	↓	NSCLC	Binds FOSL2, upregulates NAMPT, NAMPT inhibits p53, inhibits ferroptosis	Promotes cisplatin resistance	[199]
LINC01134	Nrf2/GPX4	GPX4	↓ GSH/GSSG ratio	↑	HCC	Promotes Nrf2 binding to GPX4 promoter, enhances GPX4 transcription	Confers oxaliplatin resistance	[200]
AFAP1-AS1	Wnt/β-catenin pathway	–	↑	↓	TNBC	Activates Wnt/β-catenin signaling	Promotes radioresistance	[201]

NRF2, Nuclear factor erythroid 2-related factor 2; NQO1, NAD(P)H quinone dehydrogenase 1; GR, Glutathione reductase; G6PD, Glucose-6-phosphate dehydrogenase; GCLC, Glutamate-cysteine ligase catalytic subunit; GCLM, Glutamate-cysteine ligase modifier subunit; GSTP1, Glutathione S-transferase pi 1; AGK, Acylglycerol kinase; SLC25A11, Solute carrier family 25 member 11 (mitochondrial 2-oxoglutarate/malate carrier); ccRCC, Clear cell renal cell carcinoma; PVT1, Plasmacytoma variant translocation 1; STAT3, Signal transducer and activator of transcription 3; GPX4, Glutathione peroxidase 4; OS, Osteosarcoma; HCC, Hepatocellular carcinoma; MGST1, Microsomal glutathione S-transferase 1; GPCRs, G protein-coupled receptors; NSCLC, Non-small cell lung cancer; ATO, Arsenic trioxide; TALDO1, Transaldolase 1; CRC, Colorectal cancer; PPP, Pentose phosphate pathway; NADPH, Nicotinamide adenine dinucleotide phosphate; NUTM2A-AS1, NUT family member 2A antisense RNA 1; GC, Gastric cancer; VEGFA, Vascular endothelial growth factor A; ALL, Acute lymphoblastic leukemia; NEAT1, Nuclear paraspeckle assembly transcript 1; MIOX, Myo-inositol oxygenase; ELAVL1, ELAV like RNA binding protein 1 (HuR); CASC2, Cancer susceptibility candidate 2; SOCS2, Suppressor of cytokine signaling 2; HCP5, HLA complex P5; TNBC, Triple-negative breast cancer; LSH, Lymphoid-specific helicase; GSEC, Gastric cancer survival-associated enhancer RNA; LUAD, Lung adenocarcinoma; CASC11, Cancer susceptibility candidate 11; SLC7A11, Solute carrier family 7 member 11 (xCT); GDIL, GSH Degradation Inhibiting LncRNA; CHAC1, ChaC glutathione specific gamma-glutamylcyclotransferase 1; XRN2, 5′-3′ exoribonuclease 2; MT1DP, Metallothionein 1D pseudogene; HO-1, Heme oxygenase 1; ESCC, Esophageal squamous cell carcinoma; DNMTs, DNA methyltransferases; EC, Esophageal cancer; ITGB2-AS1, Integrin subunit beta 2 antisense RNA 1; FOSL2, FOS like 2, AP-1 transcription factor subunit; NAMPT, Nicotinamide phosphoribosyltransferase; AFAP1-AS1, Actin filament associated protein 1 antisense RNA 1; NP, Nanoparticle; siRNA, Small interfering RNA; GSH, Glutathione; ROS, Reactive oxygen species; MDA, Malondialdehyde; ceRNA, Competing endogenous RNA; GSSG, Oxidized glutathione.

### 4.3 LncRNA-GSH regulation in cancer progression

LncRNAs have been reported to influence tumor progression by modulating GSH levels. Wang et al. utilized RNA sequencing to demonstrate that lncRNA XLOC_009190 regulates MGST1 through cis interactions [180]. MGST1, involved in GSH oxidation via its GPX activity, when overactivated, may lead to increased GSH consumption, thus contributing to tumor growth.

An intriguing example is SMIM26, a microprotein encoded by the lncRNA LINC00493, which exerts an anti-metastatic function in clear cell renal cell carcinoma (ccRCC) [178]. It modulates mitochondrial GSH import and respiratory efficiency, while also inhibiting the AGK-mediated AKT signaling pathway. Similarly, both LINC00493 and NUTM2A-AS1 have been shown to regulate mitochondrial GSH import and the oxidative stress response, respectively, thereby influencing cell viability, proliferation, and drug sensitivity in cancer cells [178,184,185]. Additionally, the lncRNA VPS9D1-AS1 upregulates the expression of GPX1, leading to increased intracellular GSH consumption, promoting cell proliferation, and preventing cell death in the development of acute lymphoblastic leukemia (ALL) [186].

### 4.4 LncRNA-GSH regulation in ferroptosis

By modulating genes involved in GSH metabolism, lncRNAs can either promote or inhibit tumor progression through the regulation of ferroptosis. Several studies have specifically highlighted the essential protective role of GSH in ferroptosis. He et al. discovered that ketamine induces ferroptosis by regulating the axis of lncRNA PVT1/miR-214–3p/GPX4, while inhibiting lncRNA PVT1 accelerates ferroptosis and suppresses HCC cell proliferation [187]. Moreover, the upregulation of lncRNA PVT1 has been shown to facilitate the development of osteosarcoma by activating the STAT3/GPX4 axis, thereby reducing ferroptosis and modulating GSH and ROS levels [179].

Zhang et al. revealed that lncRNA NEAT1 regulates ferroptosis by competing with miR-362–3p, leading to decreased MIOX and GSH suppression, thus increasing HCC cells’ susceptibility to iron-induced cell death [188]. Researchers also observed that hypoxia-induced HIF-1α enhances lncRNA PMAN, which increases GSH levels, stabilizes ELAVL1 cytoplasmic translocation, and enhances SLC7A11 mRNA stability, thereby reducing ferroptosis in gastric cancer peritoneal metastases [189].

Furthermore, POU6F1 was found to upregulate lncRNA CASC2, leading to reduced GSH levels through increased SOCS2-mediated ubiquitination and degradation of SLC7A11, thereby inducing ferroptosis in gastric cancer (GC) cells [190]. These findings suggest a potential therapeutic strategy targeting ferroptosis pathways for cancer treatment. In triple-negative breast cancer (TNBC), elevated lncRNA HCP5 increases GSH levels by upregulating GPX4 expression, reducing lipid ROS, and inhibiting ferroptosis, suggesting a potential therapeutic target for improved treatment outcomes [191].

Additionally, upregulated lncRNA LINC00618 binds to lymphoid-specific helicase (LSH) protein, leading to reduced SLC7A11 expression and GSH levels, heightened lipid ROS, and enhanced ferroptosis, thereby presenting a potential therapeutic target for AML treatment [192]. Researchers have also identified the lncRNA GSEC as a regulator of miRNA-101–3p and CISD1, molecules that influence GSH levels and ferroptosis, contributing to tumor progression. Jiang et al. showed that GSEC could serve as a diagnostic and therapeutic biomarker for lung adenocarcinoma (LUAD) [193]. These studies further underscore the critical role of lncRNAs in regulating GSH metabolism and modulating ferroptosis in cancer cells (Table 4).

### 4.5 LncRNA-GSH regulation in treatment resistance

LncRNAs also contribute substantially to treatment resistance through the regulation of GSH and associated detoxification pathways (Table 4). LncRNA CASC11 regulates the cystine/GSH pathway by targeting SLC7A11, which enhances GSH levels and reduces ROS [194]. CASC11 also contributes to HCC progression and modulates the efficacy of sorafenib treatment. Downregulated lncRNA SLC7A11-AS1 inhibits miR-33a-5p, resulting in increased expression of xCT and GSH levels, which reduces ROS and enhances cisplatin resistance in GC [195].

A recent work published by our team reported that a novel lncRNA, GDIL (GSH Degradation Inhibiting LncRNA), was upregulated in platinum resistant cancers [196]. LncRNA GDIL could induce chemoresistance by promoting GSH accumulation. Mechanistically, GDIL binds and re-localizes XRN2 protein to cytoplasm, where XRN2 could further degrade CHAC1 mRNA.

The increased expression of lncRNA MT1DP, which binds to miR-365a-3p and regulates the NRF2 pathway, could enhance NSCLC cells’ sensitivity to elastin-induced cell death [111]. Upregulation of LINC01419 and LINC01270 in EC promotes the methylation of GSTP1, ultimately reducing the sensitivity of EC cells to 5-fluorouracil (5-FU) [197,198]. Pep-AP, a peptide encoded by a lncRNA, sensitizes CRC cells to oxaliplatin by inhibiting TALDO1, which decreases NADPH production and GSH levels, subsequently raising ROS and promoting cell death [183]. By binding to the transcription factor FOSL2, lncRNA ITGB2-AS1 raises ROS levels and reduces GSH levels, thereby enhancing NAMPT expression. Targeting the ITGB2-AS1/FOSL2/NAMPT axis offers a potential therapeutic strategy to overcome cisplatin resistance in NSCLC [199]. Ying et al. suggested that lncRNA NUTM2A-AS1 contributes to the reduced sensitivity of GC cells to Matrine treatment [184]. Deletion of NUTM2A-AS1 in GC cells led to elevated GSH, ROS, and SOD levels.

As mentioned earlier, linc-ROR is a recently identified lncRNA that inhibits p53 protein translation, contributing to arsenic trioxide (ATO) resistance. ATO reduces GSH and SOD levels, leading to oxidative stress and p53-mediated apoptosis. Linc-ROR may enhance ATO resistance in liver cancer, while GSH could potentially alleviate the oxidative stress induced by ATO treatment [182]. LncRNA MSTRG.51053 adsorbs miR-432–5p to upregulate MGST3, influencing the GST pathway, GSH levels, ROS levels, and cisplatin resistance in NSCLC cells [181]. Silencing lncRNA LINC01134 downregulated GPX4 expression, reduced GSH levels, elevated ROS and lipid ROS levels, and promoted ferroptosis, thereby sensitizing HCC cells to oxaliplatin [200].

Radiotherapy generally increases ROS levels. The lncRNA AFAP1-AS1 has been implicated in promoting radioresistance in triple-negative breast cancer (TNBC) through the activation of the Wnt/β-Catenin signaling pathway [201]. Silencing of lncRNA AFAP1-AS1 was shown to reduce intracellular GSH levels, resulting in a synergistic reversal of resistance.

### 4.6 GSH-regulation-based lncRNA-targeted applications in cancer

The modulation of GSH by lncRNAs offers a promising approach for the discovery of new cancer treatments. Targeting lncRNAs that regulate GSH metabolism could enhance the effectiveness of existing treatments and overcome resistance mechanisms (Table 5).

**Table 5:** Novel Applications of lncRNA Based on GSH Regulation in Cancer.

Application	lncRNA	Delivery Systems	Mechanism of Release	Mechanism of Action	Cancer	Ref
Radiotherapy sensitization	AFAP1-AS1	Reduction-responsive NPs	GSH-triggered siRNA release in tumor microenvironment	Silences AFAP1-AS1 and inhibits Wnt/β-catenin pathway, Scavenges intracellular GSH and enhances ROS-mediated radiation damage	TNBC	[201]
Immunotherapy and gene therapy	ANRIL	DTBP-3-conjugated GSH-responsive NPs	GSH-triggered DTBP-3 peptide release and siRNA delivery	Blocks TIGIT/PVR interaction and enhances NK/T cell activity, silences ANRIL and inhibits miR-203a pathway	HCC	[203]
Ferroptosis sensitization	MT1DP	FA-LPs co-delivering system	Folate receptor-mediated endocytosis in tumor cells	MT1DP stabilizes miR-365a-3p and downregulates NRF2, NRF2 suppression, decreases antioxidant defense, increases lipid ROS/Fe^2+^ and induces ferroptosis	NSCLC	[111]
Chemotherapy sensitization	GDIL	LNA-modified ASO	–	Upregulates CHAC1, promotes GSH accumulation, enhances ROS scavenge	CRC	[196]
Therapy	MALAT1	LNA gapmeR ASO	–	Silences MALAT1, upregulates KEAP1, downregulates NRF1/NRF2 and proteasome activity, Disrupts MALAT1-NRF1 positive feedback loop and induces ROS	MM	[204]

NPs, Nanoparticles; GSH, Glutathione; siRNA, Small interfering RNA; TNBC, Triple-Negative Breast Cancer; DTBP-3, Designed TIGIT-Blocking Peptide 3; TIGIT, T cell immunoreceptor with Ig and ITIM domains; PVR, Poliovirus receptor; NK, Natural Killer (cells); T cells, T lymphocytes; HCC, Hepatocellular Carcinoma; FA-LPs, Folate-modified Liposomes; NRF2, Nuclear factor erythroid 2-related factor 2; ROS, Reactive Oxygen Species; NSCLC, Non-Small Cell Lung Cancer; LNA, Locked Nucleic Acid; ASO, Antisense Oligonucleotide; KEAP1, Kelch-like ECH-associated protein 1; NRF1, Nuclear respiratory factor 1; MM, Multiple Myeloma; CRC, Colorectal Cancer; CHAC1, ChaC glutathione specific gamma-glutamylcyclotransferase 1.

Various strategies targeting lncRNA-GSH interactions have emerged in recent years, including the use of nanomaterials and other innovative approaches. For example, Chen et al. explored the combination of oxygen-enhanced sonodynamic therapy (O_2_-SDT) with nanobubbles (NBs) and low-frequency ultrasound (LFUS) for HCC treatment [202]. One study employed reduction-responsive nanoparticles to deliver siRNA targeting lncRNA AFAP1-AS1, combined with radiotherapy for the treatment of TNBC. This strategy depletes GSH and inhibits the Wnt/β-Catenin pathway [201]. Another investigation utilized reduction-responsive nanoparticles to deliver siRNA targeting lncRNA ANRIL in conjunction with immunotherapy for HCC treatment. This strategy eliminated GSH and disrupted the TIGIT/PVR signaling axis, thereby strengthening the anti-tumor immune response and inhibiting tumor growth [203]. Additionally, delivering Erastin and lncRNA MT1DP using folate-modified liposomes to enhance NSCLC cell sensitivity to ferroptosis, with MT1DP modulating the miR-365a-3p/NRF2 axis, reducing GSH, and increasing lipid ROS, which promotes ferroptosis and cancer cell death [111].

Antisense oligonucleotides (ASOs), by binding to and degrading RNA targets via RNase H, have been commercially proved as RNA-based therapeutics for a range of diseases by US Food and Drug Administration (FDA) and European Medicines Agency (EMA) [175]. Our work showed that co-administration of oxaliplatin with lncRNA GDIL-targeting ASOs demonstrated significant efficacy in inhibiting tumor regrowth across both cell-derived xenograft (CDX) and patient-derived xenograft (PDX) experimental systems [196]. In addition, LNA-gapmeR-mediated ASO inhibition of MALAT1 significantly impaired melanoma progression through proteasome inhibition and ROS elevation in both *in vitro* and *in vivo* models [204].

### 4.7 Summary

In cancer progression, lncRNAs could orchestrate GSH dynamics by regulating NRF2-targeted antioxidant genes and GPX4 expression, respectively, thereby influencing oxidative stress adaptation and tumor growth. Ferroptosis modulation emerges as a critical theme, with some lncRNAs altering GSH levels via miR-competitive sponging or ubiquitin-mediated degradation of SLC7A11, thereby dictating cellular susceptibility to iron-dependent cell death. Therapeutic resistance is similarly shaped by lncRNA activity, bolstering drug resistance through GSH-mediated ROS scavenging. The ability to target lncRNAs at different functional levels provides diverse options for innovative therapeutic strategies, including GSH-responsive nanoplatforms for lncRNA-targeted siRNA delivery and antisense oligonucleotides (ASOs) against resistance-driving lncRNAs. Despite these advances, challenges persist in elucidating tissue-specific lncRNA mechanism of action, modularity, sequence, function, structure, and optimizing delivery systems for clinical translation. Further functional studies through suitable preclinical models will validate the critical role of specific lncRNAs in cancer pathogenesis and thus facilitate the extensive exploration of such molecules as therapeutic targets for a wide range of cancers.

## 5 Other ncRNAs and their regulation of GSH metabolism

### 5.1 Circular RNAs

Circular RNAs (circRNAs) represent a unique class of covalently closed RNA molecules generated through back-splicing of pre-mRNAs. Initially dismissed as mere splicing artifacts or processing byproducts due to their apparent lack of protein-coding capacity, circRNAs have undergone a dramatic reappraisal following advances in RNA sequencing technologies and bioinformatic analyses. These technological breakthroughs have uncovered an unexpected abundance of circRNAs across diverse species. To date, thousands of evolutionarily conserved circRNAs have been identified, exhibiting dynamic expression patterns that correlate with developmental processes, physiological conditions, and various pathological states - particularly cancer. Discoveries have established circRNA research, focusing on elucidating their biological functions in GSH metabolism and cancer biology (Table 6).

**Table 6:** circRNA and snoRNA-mediated Regulation of Glutathione Metabolic Pathways in Cancer.

RNA	Target	Redox Enzymes Affected	GSH Level	Oxidative Stress	Cancer Type	Regulation	Effect	Ref
circ0060467	miR-6805	GPX4	↓ (implied by ↑ ferroptosis)	↑	HCC	Acts as a ceRNA to sponge miR-6805 and upregulates AIFM2 & GPX4	Promotes cancer progression, inhibits ferroptosis	[128]
circKIF4A	miR-1231	GPX4	↓ (implied by ↑ ferroptosis)	↑	PTC	Sequesters miR-1231 and upregulates GPX4	Promotes cancer progression	[131]
circBLNK	miR-188–3p	GPX4	↓ (implied by ↑ ferroptosis)	↑	OS	Sequesters miR-188–3p and upregulates GPX4	Promotes cancer progression, inhibits ferroptosis	[132]
circ_WASF2	miR-634	GPX4	↓ (implied by ↑ ferroptosis)	↑	PC	Sequesters miR-634 and upregulates GPX4	Promotes cancer progression, inhibits ferroptosis	[138]
circMYH9	hnRNPA2B1	PHGDH	↑	↓	CRC	recruits hnRNPA2B1 to down-regulate p53, activates PHGDH	Promotes cancer progression	[205]
crVDAC3	HSPB1(Blocks ubiquitination)	SLC7A11	↑	↓	HER2-low breast cancer	inhibits HSPB1 ubiquitination and degradation	Promotes cancer progression and T-DXd resistance, inhibits ferroptosis	[206]
cTRIP12	OGT	OGT, FTH	↑	↓	PDAC	activates O-GlcNAcylation of OGT, upregulates FTH and PD-L1	Promotes cancer progression and immune escape, inhibits ferroptosis	[207]
circADARB1	miR-615–5p	SLC7A11, GPX4	↑	↓	NPC	Acts as a ceRNA to sponge miR-615–5p and upregulates HSP90B1/SLC7A11/GPX4 pathway	Promotes cancer progression and radiotherapy resistance, inhibits ferroptosis	[208]
circP4HB	miR-1184	SLC7A11	↑	↓	LUAD	Acts as a ceRNA to sponge miR-1184 and upregulates SLC7A11	Promotes cancer progression and erastin resistance, inhibits ferroptosis	[209]
circ_0005397	PCBP2	SLC7A11, GPX4	↑	↓	PC	Recruits KAT6A to mediate H3K9 acetylation of PCBP2 promoter	Promotes cancer progression, inhibits ferroptosis	[210]
circLRFN5	PRRX2	GCH1	↓	↑	GBM	Promotes PRRX2 ubiquitination, blocks GCH1 transcription	Inhibits cancer progression, induces ferroptosis	[211]
SNORA56	28S rRNA	GCLC	↑	↓	CRC	mediates pseuduridine acidification of 28S rRNA, upregulates GCLC	Promotes cancer progression, inhibits ferroptosis	[215]

GPX4, Glutathione peroxidase 4; HCC, Hepatocellular carcinoma; PTC, Papillary thyroid carcinoma; OS, Osteosarcoma; PC, Pancreatic cancer; CRC, Colorectal cancer; PHGDH, Phosphoglycerate dehydrogenase; crVDAC3, VDAC3-derived circRNA; HSPB1, Heat Shock Protein Family B (Small) Member 1; T-DXd, Trastuzumab deruxtecan; cTRIP12, circTRIP12; PDAC, Pancreatic ductal adenocarcinoma; OGT, O-linked N-acetylglucosamine transferase; FTH, Ferritin heavy chain; NPC, Nasopharyngeal carcinoma; SLC7A11, Solute carrier family 7 member 11 (cystine/glutamate transporter, xCT); LUAD, Lung adenocarcinoma; PCBP2, Poly (RC) Binding Protein 2; GBM, glioblastoma; GCH1, GTP Cyclohydrolase 1; GCLC, Glutamate-cysteine ligase catalytic subunit; SNORA56, Small Nucleolar RNA, H/ACA Box 56; rRNA, ribosomal RNA.

The above-mentioned multiple miRNAs involved in the regulation of tumor associated GSH metabolic networks are modulated by circRNAs. These specific circRNA-miRNA regulatory axes include: circ0060467/miR-6805, circKIF4A/miR-1231, circBLNK/miR-188–3p, and circ_WASF2/miR-634 [128,131,132,138]. These circRNAs function through the competing endogenous RNA (ceRNA) mechanism, where they competitively sequestering miRNAs away from their target mRNAs and consequently blocking miRNA-mediated downstream regulation, thereby influencing GSH metabolism-related pathways.

Recent mechanistic studies have revealed several novel circRNA-mediated regulatory circuits in GSH metabolism and ferroptosis resistance. In CRC, circMYH9 drives tumor progression by binding hnRNPA2B1 to destabilize p53 mRNA, which alleviates p53-mediated suppression of phosphoglycerate dehydrogenase (PHGDH) and subsequently activates serine/glycine (SG) metabolic flux [205]. This circMYH9-SG-GSH axis represents a potential therapeutic target for CRC treatment. Similarly, in HER2-low breast cancer, VDAC3-derived circRNA (crVDAC3) confers resistance to trastuzumab deruxtecan (T-DXd) by binding HSPB1 and blocking its ubiquitination [206]. Therapeutic targeting of this axis with paritaprevir restores drug sensitivity.

Notably, multiple circRNAs have been implicated in regulating ferroptosis resistance through GSH metabolism modulation. In pancreatic cancer, circTRIP12 (cTRIP12) upregulates GSH metabolism via OGT-mediated O-GlcNAcylation and stabilization of ferritin heavy chain (FTH), while simultaneously promoting PD-L1-mediated immune evasion [207]. High cTRIP12 expression correlates with therapy resistance and poor prognosis, suggesting that PERK inhibition combined with ferroptosis inducers may overcome this resistance mechanism. Similarly, circADARB1 promotes radiotherapy resistance in nasopharyngeal carcinoma (NPC) by stabilizing SLC7A11/GPX4 through HSP90B1 upregulation [208]. Nanocarrier-delivered anti-circADARB1 siRNA synergizes with Fe^2+^ to trigger ferroptosis and radiosensitization. Additional oncogenic circRNAs regulating GSH metabolism include circP4HB and circ_0005397. CircP4HB upregulates GSH synthesis in LUAD by sponging miR-1184 to enhance SLC7A11, inhibiting ferroptosis by reducing lipid ROS [209]. This promotes tumor progression and erastin resistance. Clinically, circP4HB is a prognostic biomarker and therapeutic target, driving redox-driven chemoresistance. Circ_0005397 in pancreatic cancer, which activates PCBP2 expression through KAT6A-mediated H3K9 acetylation at the PCBP2 promoter, leading to enhanced GSH synthesis, GPX4 activation, and consequent chemoresistance [210].

Contrasting with these oncogenic circRNAs, circLRFN5 exhibits tumor-suppressive properties in glioblastoma (GBM) by promoting ubiquitin-mediated degradation of PRRX2, a transcriptional activator of GCH1 [211]. This action reduces tetrahydrobiopterin (BH4) production and impairs GSH-dependent antioxidant defenses, ultimately inhibiting GBM stem cell proliferation, tumorigenesis, and chemoresistance while enhancing ferroptosis sensitivity.

### 5.2 SnoRNA

Small nucleolar RNAs (snoRNAs) represent a conserved class of 60–300 nucleotide non-coding RNAs primarily localized in the nucleolus of eukaryotic cells, where they guide the site-specific chemical modification and processing of ribosomal RNAs (rRNAs) [212]. Beyond their canonical roles in ribosome biogenesis, emerging evidence implicates snoRNAs in diverse physiological and pathological processes, including cellular stress responses, metabolic regulation, and carcinogenesis [213]. Notably, dysregulated snoRNA expression has been increasingly recognized as a critical factor in tumor initiation, progression, and therapeutic resistance through both rRNA modification-dependent and -independent mechanisms [214].

Among cancer-related snoRNAs, SNORA56 (a box H/ACA subtype) exemplifies the expanding functional repertoire of these molecules in oncogenic pathways. Mechanistically, SNORA56 directs pseudouridylation of 28S rRNA at the U1664 position, a modification that stabilizes ribosome structure and specifically enhances the translational efficiency of GCLC mRNA [215]. This post-transcriptional regulation potently upregulates GCLC expression, driving GSH biosynthesis and conferring protection against lipid peroxidation and ferroptosis. The resultant redox adaptation promotes CRC cell proliferation, metastatic dissemination, and chemoresistance. Clinically, SNORA56 overexpression in CRC tissues and circulating plasma correlates with advanced disease stage and poor patient outcomes, while its genetic ablation sensitizes tumors to ferroptosis-inducing therapies (Table 6).

Notably, SNORA56 currently stands as the only reported snoRNA directly involved in GSH metabolic regulation in tumors. The potential involvement of other snoRNAs in GSH-related pathways remains largely unexplored, suggesting a significant gap in our understanding of snoRNA-mediated redox regulation in cancer biology.

## 6 Conclusion and future directions

Abnormal GSH metabolism is considered a metabolic hallmark of tumor cells through the modulation of key enzymes and pathway regulators. GSH metabolic reprogramming promotes tumor progression and drug resistance. In recent years, non-coding RNAs (ncRNAs) have been identified as key players in regulating various processes of GSH metabolism in diseases including cancers, making them potential targets for cancer diagnosis and therapy.

It is important to recognize that most of our understanding of ncRNAs’ role in GSH metabolism-related tumor progression and resistance is primarily based on *in vitro* models, with a lack of research in real tumor microenvironments. Therefore, one future direction is to develop models that can accurately reflect the *in vivo* environment. For instance, the currently established organoid and PDX models, are favorable tools that can more accurately recapitulate redox regulation in tumor systems.

Another important direction is the utilization of high-throughput multi-omics data, combined with more accurate and suitable model systems, which will provide valuable data for the interaction of GSH metabolism and ncRNAs. These models can be either mechanism-based knowledge models or machine-learning-based data models, offering platforms for drug target identification and the study of combined treatment effects. Significant advances have been made in machine learning approaches in drug design, including improvements in drug and target parameterization methods. These advancements are expected to offer novel, more specific, and sensitive therapeutic options, paving the way for the development of safe multimodal cancer therapies.

## CRediT authorship contribution statement

**Lu Chang:** Writing – original draft, Conceptualization. **Chao Qin:** Writing – original draft. **Jianbo Wu:** Resources. **Haoqin Jiang:** Resources. **Qianqian Xu:** Resources. **Jian Chen:** Resources. **Xiao Xu:** Resources. **Xinju Zhang:** Resources. **Ming Guan:** Writing – review & editing, Funding acquisition. **Xuan Deng:** Writing – review & editing, Writing – original draft, Investigation, Funding acquisition, Conceptualization.

## Declaration of competing interest

The authors declare that they have no known competing financial interests or personal relationships that could have appeared to influence the work reported in this paper.

## Data Availability

No data was used for the research described in the article.
